# Audio-visual experience strengthens multisensory assemblies in adult mouse visual cortex

**DOI:** 10.1038/s41467-019-13607-2

**Published:** 2019-12-12

**Authors:** Thomas Knöpfel, Yann Sweeney, Carola I. Radulescu, Nawal Zabouri, Nazanin Doostdar, Claudia Clopath, Samuel J. Barnes

**Affiliations:** 1 Laboratory for Neuronal Circuit Dynamics, Department of Brain Sciences, Imperial College, London, W12 0NN UK; 2 Department of Brain Sciences, Division of Neuroscience, Imperial College London, Hammersmith Hospital Campus, Du Cane Road, London, W12 0NN UK; 3 Department of Biomedical Engineering, Imperial College London, South Kensington Campus, London, SW7 2AZ UK; 4 UK Dementia Research Institute at Imperial College, London, UK

**Keywords:** Neural circuits, Sensory processing, Synaptic plasticity, Visual system

## Abstract

We experience the world through multiple senses simultaneously. To better understand mechanisms of multisensory processing we ask whether inputs from two senses (auditory and visual) can interact and drive plasticity in neural-circuits of the primary visual cortex (V1). Using genetically-encoded voltage and calcium indicators, we find coincident audio-visual experience modifies both the supra and subthreshold response properties of neurons in L2/3 of mouse V1. Specifically, we find that after audio-visual pairing, a subset of multimodal neurons develops enhanced auditory responses to the paired auditory stimulus. This cross-modal plasticity persists over days and is reflected in the strengthening of small functional networks of L2/3 neurons. We find V1 processes coincident auditory and visual events by strengthening functional associations between feature specific assemblies of multimodal neurons during bouts of sensory driven co-activity, leaving a trace of multisensory experience in the cortical network.

## Abstract (web summary)

Sensory stimuli usually arrive simultaneously but the neural-circuit mechanisms that combine multiple streams of sensory information are incompletely understood. The authors here show that visual-auditory pairing drives plasticity in multi-modal neuron networks within the mouse visual cortex.

## Introduction

We experience the world through many senses at once but the neural-circuit mechanisms that combine multiple streams of sensory information are incompletely understood. One view is that information from different modalities is first separately processed in primary sensory cortices and then merged in higher association areas^1,2^. Work in primary sensory areas challenges this view, finding evidence for cross-modal sensory activity^3–10^. Such cross-modal activity has been proposed to facilitate the binding and integration of multisensory events, but the neural-circuit mechanisms supporting this process are unclear^11,12^.

Our understanding of multisensory integration in the primary sensory cortices is limited. Simultaneous presentation of auditory-visual stimuli can sharpen orientation tuning in Primary Visual Cortex (V1) through activation of inhibitory circuitry^4^. However, the sharpening of visual tuning properties by sound is contingent on the features of the auditory stimulus, so that both visual response suppression^5^ and enhancement are possible during coincident audio-visual presentation^7^. How sound-driven modulation of visual activity facilitates multisensory integration in V1 has yet to be fully elucidated. One possibility is that sound-driven modulation of visual activity can drive forms of neural-circuit plasticity that support associations between streams of sensory activity. Such plasticity may involve the strengthening of functional associations between neurons that encode different features of multisensory events^11,12^. Cross-modal activity may facilitate sensory processing by enhancing neural coding strategies employed by either primary sensory modality. For example, binding of key sensory features in any of the primary sensory areas involved in cross-modal activation may build bottom up cross-sensory objects that facilitate processing of sensory scenes^13^. Alternatively, interactions between cortical and thalamic sensory processing areas may play a critical role in driving cross-modal plasticity^14,15^.

Here, we ask how neural-circuits in V1 process simultaneous streams of auditory and visual stimuli (audio-visual pairings), and whether epochs of coincident sensory activity can drive neural plasticity or adaptation. We use a combination of genetically encoded voltage (GEVI) and calcium indicators (GECI) to examine both sensory evoked responses and the network relationships between excitatory neurons in L2/3 of V1. We find repeated audio-visual pairing drives plasticity in a subset of multimodal excitatory neurons. This plasticity takes the form of an enhanced response to the presentation of a tone that has been repeatedly paired with a visual stimulus. After audio-visual pairing, network properties are modified, so that neurons with enhanced tone responses become more associated with each other. This network strengthening is greatest between neurons that exhibit sound-driven enhancement of visual activity and are therefore highly co-active during audio-visual pairing. We propose that sound-driven enhancement of visual activity is a circuit mechanism that promotes periods of co-activity between subsets of multimodal neurons leading to strengthening of specific sensory assemblies during audio-visual experience. This form of network plasticity leaves a trace of multisensory experience in the cortex.

## Results

### Evidence for multimodal neurons in L2/3 of mouse V1

**Fig. 1:**
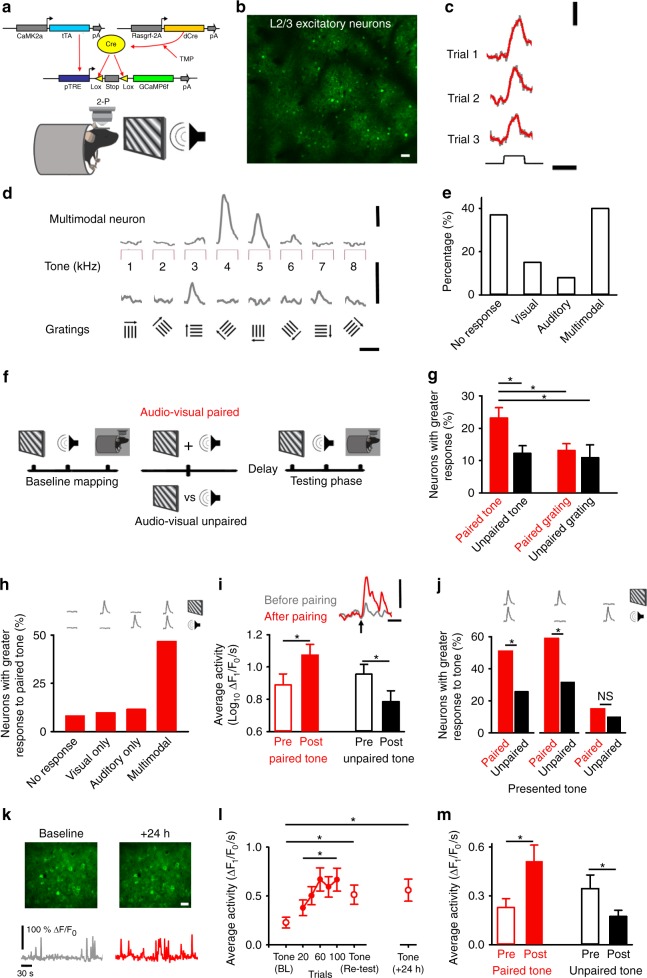
Tone-specific response enhancement at a subset of multimodal neurons after audio-visual pairing. **a**–**b** Expression of GCaMP6f in L2/3 excitatory neurons enabling *2*-P imaging in awake or lightly anaesthetised animals in V1. Scale bar: 20 µm. **c** Raw (grey) and smoothed (red) calcium responses to tone presentation. Scale bars: 50%ΔF_1_/F_0_ and 4 s. **d** Average response of multimodal neuron to tones (top) and gratings (bottom). Scale bars: 50%ΔF_1_/F_0_ and 4 s. **e** Percentage of non-responsive, visually-responsive, auditory-responsive and multimodal neurons in anaesthetised conditions. **f** Timeline depicting baseline, audio-visual pairing or repeated unpaired presentation and testing. Icons depict stimuli with resting state activity depicted by the mouse in the dark. **g** Percentage of neurons showing greater (>20% of baseline) responses to the paired (red) or unpaired (black) tone or grating after audio-visual pairing. **h** Neurons with an increased response to the paired tone vs. baseline sensory response profiles. Icons depict response to stimuli. **i** Average calcium activity (ΔF_1_/F_0_/s) following Log_10_ transformation for multimodal neurons to the paired (red) or unpaired (black) tone before (open) or after (filled) paired and unpaired trials. Inset: response to paired tone before (grey) and after (red) pairing. Scale bars: 50%ΔF_1_/F_0_ and 4 s. **j** Percentage of multimodal neurons showing an enhanced (>20%) tone response to either the paired (red) or unpaired (black) tone. Neurons grouped by baseline sensory response profiles to separate presentation of paired auditory and visual stimuli. Responsive to, (Left) paired visual and auditory stimuli, (Middle) paired visual stimuli only and (Right) paired auditory stimuli only. Icons depict visual/auditory baseline response. **k** V1 region and responses during baseline (left) and 24-h after pairing (right) in awake animals. Scale bars: 20 µm, 100%ΔF_1_/F_0_ and 30 s. **l** Average calcium (ΔF_1_/F_0_/s) response of multimodal neurons to pairing in awake animals. Open circles are tone presentation in the baseline (BL), re-testing and testing 24-h after pairing. Calcium response to pairing stimuli is shown as filled circles. **m** Average calcium activity (ΔF_1_/F_0_/s) for paired tone before (red, open) and after (red, filled) pairing and for unpaired tone (black bars). Figure 1b–j uses 332 neurons from six regions across six animals in light anaesthetic conditions and Fig. 1k–m uses 408 neurons from four regions across four animals in awake conditions. In all panels, *p < 0.05 (see Table 1 and associated Supplementary Table 1). Error bars: mean and ± S.E.M. Source data are provided as a Source Data file.

Neurons in the primary sensory cortex that respond to multiple sensory modalities may play a role in binding multisensory information^13^. To test for the presence of such neurons in V1 we measured baseline responses to separate presentation of auditory (tones: 1–8 kHz) and visual stimuli (drifting gratings: eight orientations) (Fig. 1). Using an inducible intersectional genetic approach (see Methods), we generated mice sparsely expressing GCaMP6f in a subset of L2/3 excitatory neurons (Fig. 1a). We then used 2-photon (2-P) microscopy to image neuronal calcium activity in V1 of these animals under either light anaesthesia or in awake conditions (see Methods) (Fig. 1a–d, k–m).

Sound presentation elicited responses in V1^4,5,7^ **(**Fig. 1c**)**. Around 40 % (133/332) of neurons were multimodal, exhibiting responses during presentation of both auditory and visual stimuli (Fig. 1d, e, Fig. Supplementary Fig. 1d inset). The remaining neurons were either unresponsive to both stimuli (37 %, 122/332) or responsive to visual (15 %, 51/332) or auditory (8 %, 26/332) stimuli alone **(**Fig. 1e**)**. Some multimodal neurons exhibited a best tone response, which we quantified by normalising response amplitudes to the best response and estimating tuning sharpness using a linear fit^16^ (see Methods; Fig. 1c, d and Supplementary Fig. 1a). Using this approach, we found around one third of multimodal neurons were selective to tone frequency (35 %, 43/133) (Supplementary Fig. 1a). Multimodal neurons had typical visual response properties that were similar to those of visually responsive neurons (Orientation Selectivity Index: OSI > 0.5 = 61 %, 81/133) (Supplementary Fig. 1b–d) and in line with data from previous reports^17^. We found no obvious correlation between the auditory (best response frequency) and visual (preferred orientation) sensory response properties of multimodal neurons **(**Supplementary Fig. 1e**)**. Nor did we find evidence for spatial clustering of auditory feature selectivity, so that the average distance between neurons with the same best tone response was similar to that between neurons with different best tone responses (Supplementary Fig. 1f). Our results suggest that a subset of excitatory neurons in L2/3 of V1 are multimodal, being responsive to both auditory and visual stimuli^4,7^.

### Audio-visual pairing modifies sound-evoked responses

We next asked whether audio-visual experience can modify the response properties of multimodal neurons. To answer this question, we compared activity during ‘paired’ and ‘unpaired’ audio-visual stimulation trials. For paired audio-visual trials, we simultaneously and repeatedly presented a specific pairing of auditory and visual stimuli (which were randomly selected: see Methods; Fig. 1f). For unpaired trials, a distinct set of auditory and visual stimuli different to those in the paired condition were separately presented (Fig. 1f). The number of stimulus presentations was matched between the paired and unpaired conditions (Fig. 1f). Therefore, apart from the chosen stimuli, the only difference between the paired and unpaired conditions was the relative timing of the presented audio-visual stimuli. After interleaved trials of the paired or unpaired conditions, we re-tested the response properties of neurons by comparing to our baseline mapping phase (Fig. 1f).

We found that audio-visual pairing increased the response of some neurons to the paired tone (Fig. 1g). In contrast, very few neurons showed enhancement to either the unpaired tone or the paired and unpaired visual stimuli (Fig. 1g). When we examined the functional characteristics of those neurons with an enhanced response to the paired tone, we found they were typically multimodal (enhanced response to paired tone: multimodal neurons = 46.6% 62/133) (Fig. 1h). As a result, the average activity of multimodal neurons increased after pairing (relative to baseline) in response to the paired tone (Fig. 1i, Supplementary Fig. 1g). In contrast, unpaired tone trials appeared to drive adaptation-like changes at multimodal neurons, which reduced their average activity to the unpaired tone (Fig. 1i). We found no change in the average response of multimodal neurons to the paired or unpaired visual stimulus after pairing (Supplementary Fig. 1h). Nor did we observe any difference in the orientation tuning between multimodal neurons that exhibited an enhanced tone response after audio-visual pairing and those that did not (Supplementary Fig. 1h inset). Neurons characterised as functionally silent or responsive to only visual or auditory stimulation showed little change in their responses to the paired tone (enhanced response: non-responsive = 8.2 %, 10/122; visual only = 9.8 %, 5/51; auditory only = 11.5 %, 3/26) (Fig. 1h). Hence, coincident audio-visual pairing selectively modified the cross-modal response properties of multimodal neurons in V1 to the paired tone.

### Enhanced tone response is driven by paired visual stimulus

We next asked why only a subset of multimodal neurons expresses enhanced tone responses after audio-visual pairing. We split multimodal neurons into three groups based on their properties during baseline mapping (see Methods): (1) neurons responsive to both the paired visual and auditory stimuli, (2) neurons responsive to the paired visual stimulus and (3) neurons responsive to the paired auditory stimulus (Fig. 1j). Approximately half of the neurons that responded to both the visual and auditory stimulus (during baseline) exhibited a strengthened response to the paired tone when re-tested after audio-visual pairing (Fig. 1j). These neurons also exhibited a sharpening of auditory tuning following pairing (Supplementary Fig. 1j). Around two thirds of the neurons that responded to the visual but not the auditory stimulus during baseline testing later exhibited a tone response but did not show a clear sharpening of the auditory tuning curve (Fig. 1j and Supplementary Fig. 1j). Of the neurons that responded to the auditory but not the visual stimulus very few showed tone-specific strengthening after audio-visual pairing (Fig. 1j). Thus, for multimodal neurons, a key component in the development of enhanced tone response after audio-visual pairing is activity driven by the paired visual stimulus.

Behavioural state modifies the underlying network dynamics that process sensory experience^18^. To address this issue, we repeated our experiments in awake behaving animals using a viral construct to label L2/3 excitatory neurons with both GCaMP6 and a red fluorescent structural marker (Fig. 1k) (see Methods). The majority of neurons (187/212, 88.2 %) defined as multimodal under conditions of light anaesthesia were also defined as multimodal in awake imaging conditions. Furthermore, the percentage of cells responding to different sensory stimuli (non-responsive 36.7 %, visual 14.2 %, auditory 9.8% and multimodal 39.2 %) was similar to measurements made in lightly anaesthetised animals (see Table 1). Audio-visual pairing resulted in similar responses at multimodal neurons to those seen under light anaesthesia (Fig. 1l, m). Responses to the paired tone strengthened following pairing (Fig. 1l, m), whilst responses to the unpaired tone weakened after repeated presentation (Fig. 1m). The co-expression of a structural marker allowed us to longitudinally track the same neurons across multiple imaging sessions spanning days (Fig. 1l). We used this approach to test the tenacity of pairing driven plasticity and found the strengthened tone responses at multimodal neurons were still present 24 h after pairing **(**Fig. 1l). However, in keeping with the adaptation-like changes seen following repeated tone presentation (Fig. 1i, m), strengthened tone responses could be weakened by repeated presentation of the tone stimulus in isolation (Supplementary Fig. 1l). Our results suggest that audio-visual pairing can leave a lasting functional trace of sensory experience in the cortex.

**Table 1:** Statistical comparisons for Fig. 1, related to Fig. 1.

Panel	Comparison	Test	Centre and dispersion or fit	*p* value	*N* value
1e	Percentage of neurons with response to stimulation in awake vs anaesthetised conditions	Chi-Squared test	Non-responsive Awake = 150/408, 36.7 % Anaesthetised = 122/332, 36.7 %, Visual Awake = 58/408, 14.2 % Anaesthetised = 51/332, 15.4 % Auditory Awake = 40/408, 9.8 % Anaesthetised = 26/332, 7.8 %, Multimodal Awake = 160/408, 39.2 % Anaesthetised = 133/332, 40.0 %	*p* = 0.946 *p* = 0.784 *p* = 0.467 *p* = 0.933	Ana. 332 neurons Awake 408 neurons
1g	Percentage of neurons showing an enhanced response ( > 20 % of baseline) after pairing	One-Way ANOVA With Holm-Šidák	Greater response to: Paired Tone = 23 ± 3 % Unpaired Tone = 12 ± 2 % Paired Grating = 13 ± 2% Unpaired Grating = 11 ± 4 %	P-tone Vs U-tone *p* = 0.024 P-tone Vs P-Grat *p* = 0.037 P-tone Vs U-Grat *p* = 0.018	332 neurons
1h	Neurons showing an enhanced response after pairing by response type	Descriptive	No Response = 10/122, 8.2 % Visual = 5/51, 9.8 % Auditory = 3/26, 11.5 % Multimodal = 62/133, 46.6 %	N/A	332 neurons
1i	Average activity of multimodal neurons in response to the paired tone: Pre vs Post pairing	One-Way Repeated Measures ANOVA	Pre = 0.88 ± 0.07 Post-pairing = 1.1 ± 0.07 Average activity (ΔF_1_/F_0_/s) following Log_10_ transformation	*p* = 0.021	133 neurons
1i	Average activity of multimodal neurons in response to the unpaired tone; Pre vs Post pairing	One-Way Repeated Measures ANOVA	Before = 0.95 ± 0.06 After = 0.78 ± 0.07 Average activity (ΔF_1_/F_0_/s) following Log_10_ transformation	*p* = 0.037	133 neurons
1j	Percentage of cells more responsive to paired or unpaired tone for different multimodal neuron populations	z-test of proportions	Responds to both paired visual and auditory stimulus Paired tone = 51 %, 22/43 Unpaired tone = 26 %, 11/43	*p* = 0.027	43 neurons
1j	Percentage of cells more responsive to paired or unpaired tone for different multimodal neuron populations	z-test of proportions	Responds to paired visual but NOT auditory stimulus Paired tone = 59 %, 26/44 Unpaired tone = 32 %, 14/44	*p* = 0.019	44 neurons
1j	Percentage of cells more responsive to paired or unpaired tone for different multimodal neuron populations	z-test of proportions	Responds to paired auditory but NOT visual stimulus Paired tone = 15 %, 3/20 Unpaired tone = 10 %, 2/20	*p* = 1.000	20 neurons
1 l,m	Δ in response of multimodal neurons to paired tone before and after pairing in awake mice	One-Way ANOVA With Holm-Šidák	Tone BL = 0.23 ± 0.06 Tone re-test = 0.51 ± 0.10 Tone re-test (+24 hr) = 0.56 ± 0.11 (Average calcium activity (ΔF_1_/F_0_/s)	Tone BL vs Tone re-test *p* = 0.021 Tone BL vs Tone re-test ( + 24 hr) *p* = 0.022	160 neurons
1 l	Δ in response of multimodal neurons to repeated audio-visual presentation in awake mice	Paired *t*-test	Pairing trials (15–20) = 0.38 ± 0.08 vs Pairing trials (95–100) = 0.67 ± 0.12 (Average calcium activity (ΔF_1_/F_0_/s)	*p* = 0.037	160 Neurons
1 m	Δ in response of multimodal neurons to paired tone or unpaired tone before and after repeated presentation in awake mice	*t*-test	Tone pre = 0.23 ± 0.06 vs Tone post = 0.51 ± 0.10 Unpaired tone BL = 0.35 ± 0.08 vs Unpaired tone re-test = 0.17 ± 0.04 (Average calcium activity (ΔF_1_/F_0_/s)	*p* = 0.012 *p* = 0.032 (one-tailed)	160 neurons

The data for Fig. 1 uses 332 neurons over 12 experiments taken from six cortical regions across six animals in light anaesthetic conditions and 408 neurons from four cortical regions across four animals in awake conditions

### Audio-visual pairing modifies subthreshold responses in V1

**Fig. 2:**
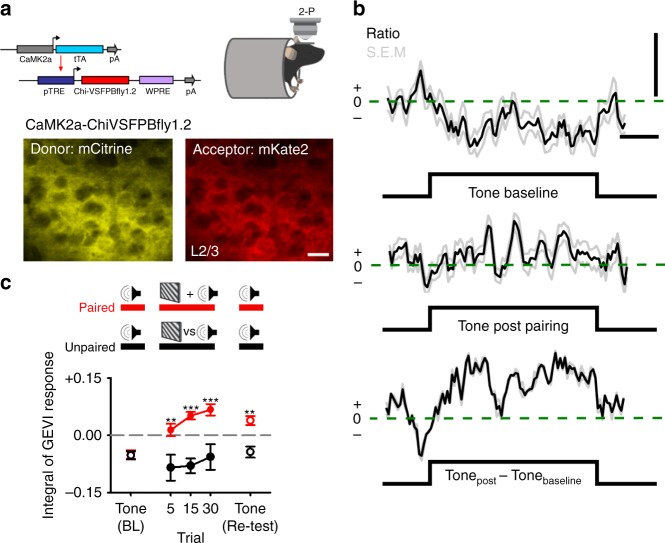
Audio-visual pairing results in a tone-specific reduction in subthreshold hyperpolarisation. **a** (Top, left) Schematic of intersectional genetic approach for the expression of Chi-VSFPBfly1.2 at excitatory (CaMK2A) cortical neurons. (Top, right) Cartoon depicting 2-P imaging of CaMK2A-Chi-VSFPBfly1.2 expressing mouse under anaesthetised conditions. (Bottom) Example region taken from L2/3 of V1 in a CaMK2A-Chi-VSFPBfly1.2 expressing adult mouse showing FRET channels for the donor (mCitrine–left) and acceptor (mKate2–right). Scale bar: 10 µm. **b** Example ratio traces (Ratio = Acceptor: mKate2/Donor: mCitrine) showing change in tone response following pairing. (Top) Response to tone presentation during the baseline mapping phase. (Middle) Response to the same tone following audio-visual pairing. (Bottom) Difference between the tone presentation before and after audio-visual pairing. Black line shows the average ratio response across nine cortical regions and grey lines show the S.E.M. of responses. Scale bars are 1 % and 1 s. **c** Population level cross-modal plasticity measured with CaMK2A-Chi-VSFPBfly1.2 following repeated audio-visual pairing (red) and repeated unpaired tone presentation (black). The data for Fig. 2a–c uses 356 regions taken from five animals under anaesthetised conditions. For all panels, **p < 0.01, ***p < 0.001 (see Table 2 and associated Supplementary Table 2). Error bars: mean and ± S.E.M. Source data are provided as a Source Data file.

Our calcium imaging experiments found a considerable proportion of multimodal neurons developed a response to presentation of the paired tone after audio-visual pairing (Fig. 1j). Prior to pairing, these neurons could be synaptically activated by sound below the threshold for action potential generation or might undergo net inhibition during sound presentation^5^. In both cases, the subthreshold auditory response would not be initially detected by calcium imaging but could be unmasked following audio-visual pairing. We examined the sub-threshold and hyperpolarising component of cross-modal activation in vivo using 2-P imaging to measure voltage responses from mice expressing the GEVI Chimeric-VSFPBfly1.2 in excitatory neurons of V1 (Fig. 2a). We repeated our audio-visual pairing experiment (Fig. 1f) and analysed voltage responses using a previous method which takes the spatial temporal average of the GEVI signal over multiple regions of interest (ROI) in each imaged animal (see Methods)^19–21^ (Fig. 2a and Supplementary Fig. 2a–c).

**Table 2:** Statistical comparisons for Fig. 2, related to Fig. 2.

Panel	Comparison	Test	Centre and dispersion or fit	*p* value	*N* value
2c	Response to paired vs unpaired tone presentation during baseline	One-Way ANOVA With Holm-Šidák Post-hoc testing	Baseline: Paired = −0.05 ± 0.01 vs Unpaired = −0.05 ± 0.01 Integral of GEVI response	*p* = 0.902	A–V pairing *n* = 176 regions with the average of 5 trials Repeated tone *n* = 180 regions with the average of 5 trials
2c	Response to coincident audio-visual pairing vs unpaired tone	One-Way ANOVA With Holm-Šidák Post-hoc testing	Trial 5: Paired = 0.01 ± 0.02 vs Unpaired = −0.09 ± 0.03 Trial 15: Paired = 0.05 ± 0.01 vs Unpaired = −0.08 ± 0.02 Trial 30: Paired = 0.07 ± 0.02 vs Unpaired = −0.06 ± 0.03 Integral of GEVI response	Trial 5: *p* = 0.003 Trial 15: *p* < 0.001 Trial 30: *p* < 0.001	A–V pairing *n* = 176 regions with the average of 5 trials Repeated tone *n* = 180 regions with the average of 5 trials
2c	Response to paired vs unpaired tone presentation during re-testing	One-Way ANOVA With Holm-Šidák Post-hoc testing	Re-testing: Paired = 0.04 ± 0.01 vs Unpaired = −0.04 ± 0.02 Integral of GEVI response	*p* = 0.002	A–V pairing n = 176 regions with the average of 5 trials Repeated tone *n* = 180 regions with the average of 5 trials

The data for Fig. 2 uses 356 regions taken from five animals.

Prior to pairing, we observed a net hyperpolarising response to tones (Fig. 2c). Together with our calcium imaging experiments, and the findings of others, this net hyperpolarisation suggests that sound drives a complex mix of both inhibition and supra-threshold activation in V1^4,7^. Coincident audio-visual pairing resulted in depolarising responses that increased across multiple trials (Fig. 2c). When we re-tested tone responses after pairing we observed a net depolarisation when the paired tone was presented on its own, this was not the case for presentation of the unpaired tone (Fig. 2b, c). The tone-specific shift from hyperpolarisation to depolarisation suggests that specific neural-circuits are strengthened by audio-visual pairing, and that these modifications alter the subthreshold sound-evoked signal in V1.

### Multimodal neurons share functional subnetworks

Changes in subthreshold signalling suggest synaptic adaptation may occur during audio-visual pairing. To investigate further, we explored the neural-circuit organisation of multimodal neurons by probing local network associations^22–25^. L2/3 excitatory neurons in V1 are known to share small subnetworks organised by visual feature selectivity^23^. Because activity driven by the paired visual stimulus was a key component in the development of enhanced tone response after audio-visual pairing (Fig. 1j), we reasoned that the multimodal neurons which exhibit tone plasticity in our experiment may exhibit such local circuit organisation. Functional cortical networks can be studied using pairwise correlations of calcium signals^23,26^. These correlations are thought to reflect either synaptic connections between cells and/or common inputs^22,23,25,27,28^.

**Fig. 3:**
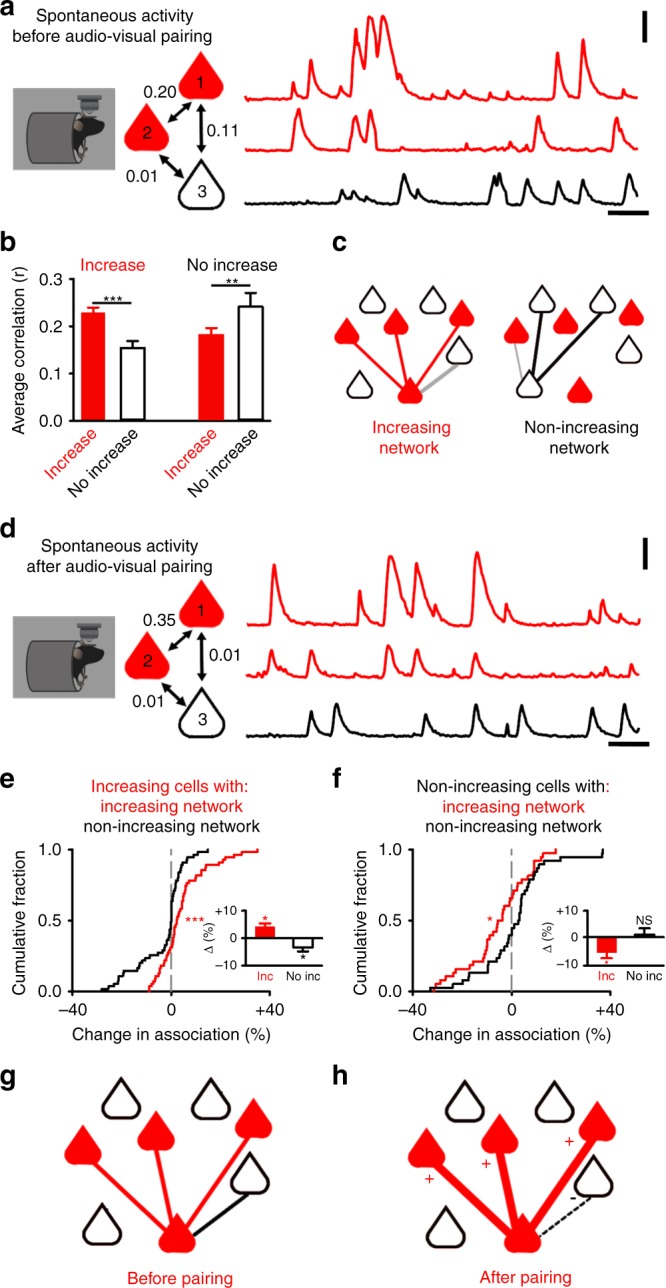
Multimodal subnetworks exhibit strengthening and weakening of functional associations. **a**, **d** Correlations between multimodal neurons during spontaneous activity before (**a**) and after (**d**) pairing. Example shows multimodal neurons that either have an increased tone response after pairing (red, filled) or do not (black, open). Scale bar: 20 s and 100 %ΔF_1_/F_0_. **b** Average correlation strength (r) prior to audio-visual pairing between multimodal neurons that either: have an increased response to the paired tone after pairing (left) or do not (right). Average correlation value in each case is with other neurons that either: have increased tone responses (red, filled) or do not (black, open). **c** Schematic showing multimodal networks to which a neuron with increased (Left: red, filled) or not increased (Right: black, open) responses to the paired tone may belong. Red line represents associations between neurons with an increased tone response. Black line represents associations between neurons that do not have an increased tone response. Grey line indicates associations between neurons from different groups. **e**, **f** Change in the percentage of the correlation coefficient (out of summed total of all correlation coefficients) attributable to multimodal neurons with different responses to pairing. For neurons with increased (**e**) or non-increased (**f**) responses, the percentage change in total correlation coefficient attributable to associations with other increasing neurons (red) or other neurons that do not increase (black). The grey dashed line in **e**, **f** depicts 0% change. Inset: average percentage change for increasing (**e**) and non-increasing (**f**) cells. **g**, **h** Schematic of a multimodal subnetwork to which a neuron that has an enhanced tone response (red, filled) may belong to, before (**g**) and after (**h**) audio-visual pairing. Neurons which do not show an enhanced response are shown as black and open. Red lines represent strong functional associations, which increase in strength (as indicated by the thickness of the line and addition sign) whilst black line represents weaker functional associations, which weaken (as indicated by the dashed black line and subtraction sign). Figure 3a–f uses 103 neurons taken from five cortical regions across five animals in anaesthetised conditions. For all panels, *p < 0.05, **p < 0.01, ***p < 0.001 (see Table 3 and associated Supplementary Table 3). Error bars: mean and ± S.E.M. Source data are provided as a Source Data file.

We measured correlations between neuronal calcium signals during periods of spontaneous resting state activity (Fig. 3a) and sensory stimuli (Supplementary Fig. 3c) prior to audio-visual pairing. We found multimodal neurons with common visual feature selectivity did form subnetworks (Supplementary Fig. 3a). This organisation meant that multimodal neurons that would later increase their response to the paired tone were more likely to be correlated with each other (Fig. 3a–c, Supplementary Fig. 3c). Conversely, cells that did not exhibit an increased tone response after pairing were more strongly correlated with other cells that also did not increase their response (Fig. 3b, Supplementary Fig. 3c). Our results suggest that multimodal neurons that undergo similar forms of plasticity share functional subnetworks (Fig. 3c).

**Table 3:** Statistical comparisons for Fig. 3, related to Fig. 3.

Panel	Comparison	Test	Centre and dispersion or fit	*p* value	*N* value
3b	For increasing neurons with increasing network vs with non-increasing network during baseline	Repeated measures ANOVA	For increasing neurons with increasing network = 0.23 ± 0.01 vs For increasing neurons with non-increasing network = 0.15 ± 0.01 (Average correlation coefficient (r))	*p* < 0.001	65 Increasing 38 Non-increasing neurons
3b	For non-increasing neurons with increasing network vs with non-increasing network during baseline	Repeated measures ANOVA	For non-increasing neurons with increasing network = 0.18 ± 0.02 vs For non-increasing neurons with non-increasing network = 0.24 ± 0.03 (Average correlation coefficient (r))	*p* = 0.007	65 Increasing 38 Non-increasing neurons
3e Main	For increasing neurons percentage change of total correlation attributable to increasing network vs non-increasing network	*t*-test	Increasing network = + 4.2 ± 1.3 % vs non-increasing network = −3.4 ± 1.3 %	*p* < 0.001	55 Increasing 38 Non-increasing neurons
3e inset	For increasing neurons percentage of total correlation attributable to increasing network Pre-pairing vs Post-pairing, percentage difference shown in inset	Repeated measures ANOVA	Pre-Pairing = 37 ± 3 % vs Post-pairing = 41 ± 3 %,	*p* = 0.019	55 Increasing 38 Non-increasing neurons
3e inset	For increasing neurons percentage of total correlation attributable to non-increasing network Pre-pairing vs Post-pairing, percentage difference shown in inset	Repeated measures ANOVA	Pre-Pairing = 15 ± 2 % vs Post-pairing = 11 ± 2 %,	*p* = 0.010	55 Increasing 38 Non-increasing neurons
3f Main	For non-increasing neurons percentage change of total correlation attributable to increasing network vs non-increasing network	*t*-test	Increasing network = −5.7 ± 2.1 % vs non-increasing network = 1.0 ± 2.2 %	*p* = 0.031	55 Increasing 38 Non-increasing neurons
3f inset	For non-increasing neurons percentage of total correlation attributable to increasing network Pre-pairing vs Post-pairing, percentage difference shown in inset	Repeated measures ANOVA	Pre-Pairing = 30 ± 2 % vs Post-pairing = 24 ± 2 %,	*p* = 0.011	55 Increasing 38 Non-increasing neurons
3f inset	For non- increasing neurons percentage of total correlation attributable to non-increasing network Pre-pairing vs Post-pairing, percentage difference shown in inset	Repeated measures ANOVA	Pre-Pairing = 25 ± 2 % vs Post-pairing = 26 ± 3 %,	*p* = 0.636	55 Increasing 38 Non-increasing neurons

The data for Fig. 3 uses 103 neurons taken from five cortical regions across five animals

### Selective strengthening of functional multimodal subnetworks

Theoretical work has proposed that multimodal neurons may exhibit network level plasticity resulting in functional assemblies that encode multisensory experience^11,12^. To investigate this prediction, we asked whether multimodal neurons underwent network level changes following audio-visual pairing. To estimate functional network changes, we measured the fraction of all pairwise correlation coefficients attributable to neurons with increasing or non-increasing tone responses for each multimodal neuron (see Methods). We again used periods of spontaneous (Fig. 3d, Supplementary Fig. 3a, b) or sensory driven (Supplementary Fig. c, d) activity and compared recordings collected before and after audio-visual pairing (Fig. 3e, f, Supplementary Fig. 3d).

Multimodal neurons with increased tone responses became more associated with each other after audio-visual pairing (Fig. 3e, inset and Supplementary Fig. 3d) and less associated with neurons without increased responses (Fig. 3e, f, inset and Supplementary Fig. 3d). Functional associations between neurons that did not exhibit an increased tone response were unaffected by audio-visual pairing (Fig. 3f, inset and Supplementary Fig. 3d). Our results could not be explained by differences in spontaneous activity levels, which were similar for multimodal neurons that either exhibited an increased tone response, or did not, after audio-visual pairing (Supplementary Fig. 3b). Our data suggest that audio-visual experience can induce specific network level adaptation, so that small assemblies of multimodal neurons are selectively strengthened (Fig. 3g, h).

### Simulation of multimodal network plasticity

**Fig. 4:**
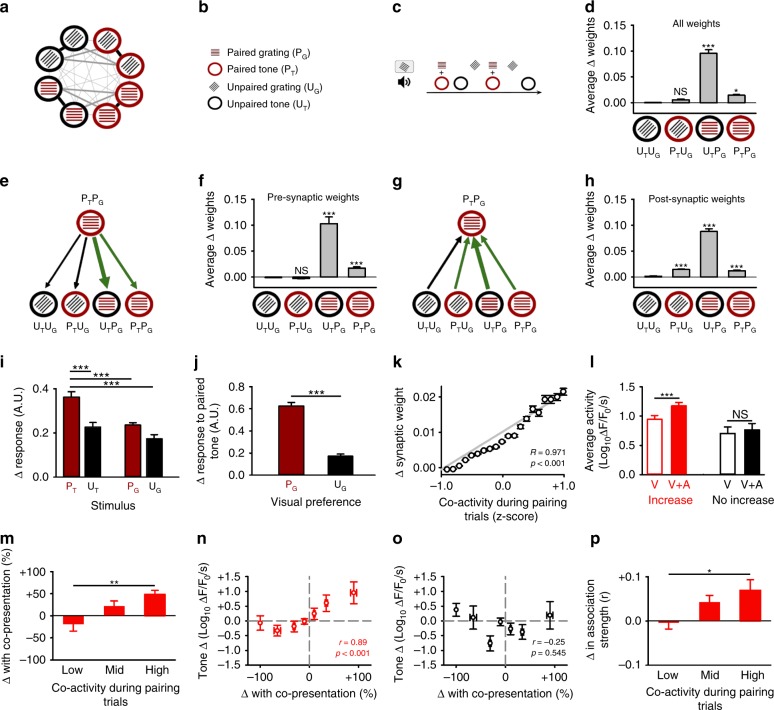
Periods of co-activity predict multimodal network plasticity. **a**–**c** Schematic of simulation with key (**b**) and plasticity protocol (**c**). Width of grey lines indicate strength of connections following development (**a**). **d** Average change in all synaptic weights at multimodal neurons (preferring paired tone and grating–P_T_P_G_) icons as in **b**. **e**–**h** Main plasticity changes in the simulation (**e**, **g**). Thickness of arrow denotes average change in strengthened (green) and unchanged (black) synaptic weights. Change in synaptic weights from (**e**, **f**) or onto (**g**, **h**) multimodal neurons (preferring both the paired tone and grating - P_T_P_G_) with other cells. **i**) Change in responses at multimodal neurons after pairing to presentation of stimuli. **j** Change in response to paired tone for multimodal neurons tuned to either the paired or unpaired visual grating. **k** Co-activity between multimodal neurons during pairing and later synaptic strengthening. **l** Average calcium activity (ΔF_1_/F_0_/s following Log_10_ transformation) for multimodal neurons that either exhibit an increased response (left, red) or do not (right, black) following audio-visual pairing. Bars show the average activity in response to either the paired visual stimuli alone (open) or the coincident presentation of the paired visual stimuli with the paired tone (filled). **m**, **p** Change in average activity when sound is presented with visual stimuli (**m**) or change in correlation coefficient between cell pairs after pairing (**p**) for cell pairs with either low (<25 %), medium (25–50 %) or high (>50 %) co-activity during audio-visual pairing trials. **n**, **o** Correlation plots for all multimodal neurons. The x-axis gives the change in activity between the response to auditory and visual stimuli when presented separately (summed) or simultaneously. The y-axis gives the change in calcium activity (ΔF_1_/F_0_/s following Log_10_ transformation baseline vs re-testing phase) in response to **n** paired or **o** unpaired tone presentation. Figure 4a–k uses 50 networks of 200 neurons with results averaged across all network simulations. Experimental data in Fig. 4l–p uses 332 neurons taken from six cortical regions across six animals in anaesthetised conditions. For all panels, *p < 0.05, **p < 0.01, ***p < 0.001 (see Table 4 and associated Supplementary Table 4). Error bars, mean and ± S.E.M. Source data are provided as a Source Data file.

To gain mechanistic insight into the network interactions that may support plasticity between multimodal neurons we used a computational approach based on BCM plasticity rules^29,30^. We generated networks of multimodal neurons by seeding nodes in a recurrent model with a preference for feedforward sensory input representing visual stimuli, auditory stimuli or both. To reflect V1 circuitry, we biased our network in favour of visual input and used a developmental phase to establish subnetworks of neurons organised by visual feature selectivity (see Methods) (Fig. 4a, Supplementary Fig. 3a, Supplementary Fig. 4a–c). We then simulated our audio-visual pairing experiment by repeatedly presenting paired or unpaired auditory and visual stimuli to the network (Fig. 4a–c). The stimulus pairing protocol caused a substantial strengthening between specific subsets of multimodal neurons (Fig. 4d–h). Unsurprisingly, the Hebbian plasticity rules in our network meant that neurons tuned to both the paired grating and the paired tone were co-active during pairing and therefore strengthened their associations (Fig. 4d–h). The synapses between these neurons were among some of the strongest prior to pairing (after development) and therefore close to saturation, as a result, these synapses only showed small positive changes after pairing (Fig. 4d–h). However, neurons tuned to the paired visual grating, but not the paired tone, developed strong connections in both directions with neurons that preferred both the paired tone and grating (Fig. 4e–h). This synaptic strengthening occurred because the simultaneous presentation of grating and tone drove the postsynaptic neuron’s firing rate above the threshold for potentiation (*θ*_*i*_). Strengthening the Pre → Post association between these neurons eventually led to later reciprocal strengthening (Fig. 4e–h). In contrast, presenting auditory or visual stimuli separately (unpaired) was not sufficient to substantially exceed the threshold for potentiation. Thus, a key factor in driving plasticity in our simulation was that tone presentation enhanced visual activity above the threshold for potentiation. When we examined the response profiles of simulated multimodal neurons after pairing, we found an enhanced response to the paired tone (Fig. 4i). This enhanced tone response was greatest at those neurons with a preference for the paired visual stimulus prior to pairing (Fig. 4j). The enhanced tone response occurred because neurons responsive to the paired grating were recruited by multimodal neurons to the paired tone network (Fig. 4e–h). This result is in agreement with our in vivo data, where we find tone plasticity is greatest at multimodal neurons responsive to the paired grating prior to pairing (Fig. 1j). Taken together, the results of our simulation suggest that coincident audio-visual stimulation increases the firing rate of some multimodal neurons, which in turn increases periods of co-activity between cell pairs and leads to selective network strengthening (Fig. 4k). This strengthening recruits neurons responsive to the paired visual stimulus to the paired tone network and thus enhances their response to the paired tone (Fig. 4e–j).

**Table 4:** Statistical comparisons for Fig. 4, related to Fig. 4.

Panel	Comparison	Test	Centre and dispersion or fit	*p* value	*N* value
4d	Δ in all synaptic weights at multimodal neurons P_T_P_G_ (Average change in synaptic weights after pairing)	One-Way ANOVA	U_T_U_G_ = 0.001 ± 0.001 vs P_T_U_G_ = 0.010 ± 0.001 U_T_P_G_ = 0.096 ± 0.007 P_T_P_G_ = 0.015 ± 0.001	*p* = 0.286 *p* < 0.001 *p* = 0.011	Average of 50 simulations
4f	Δ synaptic weights from multimodal neurons P_T_P_G_ (Average change in synaptic weights after pairing)	One-Way ANOVA on Ranks	U_T_U_G_ = 0.001, −0.001–0.001 vs P_T_U_G_ = −0.001, −0.003–0.001 U_T_P_G_ = 0.075, 0.033–0.150 P_T_P_G_ = 0.013, 0.003–0.025	*p* = 1.000 *p* < 0.001 *p* < 0.001	Average of 50 simulations
4h	Δ synaptic weights onto multimodal neurons P_T_P_G_ (Average change in synaptic weights after pairing)	One-Way ANOVA on Ranks	U_T_U_G_ = 0.001, 0.001–0.003 vs P_T_U_G_ = 0.013, 0.010–0.019 U_T_P_G_ = 0.083, 0.063–0.111 P_T_P_G_ = 0.010, 0.005–0.019	*p* < 0.001 *p* < 0.001 *p* < 0.001	Average of 50 simulations
4i	Change in response of multimodal neurons after pairing to different stimuli	One-Way ANOVA	P_T_ = 0.36 ± 0.02 vs U_T_ = 0.23 ± 0.02 P_G_ = 0.24 ± 0.01 U_G_ = 0.17 ± 0.02 (Change in activity A.U.)	*p* < 0.001 *p* < 0.001 *p* < 0.001	Average of 50 simulations
4j	Change in response to paired tone for multimodal neurons preferring the paired or unpaired visual stimuli	*t*-test	P_G_ = 0.63 ± 0.03 vs U_G_ = 0.17 ± 0.02 (Change in activity A.U.)	*p* < 0.001	Average of 50 simulations
4k	Correlation between co-activity during pairing trials and change in synaptic weight	Linear Regression	*R* = 0.971	*p* < 0.001	Average of 50 simulations
4l	For increasing multimodal neurons avg response to visual stimulus vs visual and auditory stimulus	One-Way Repeated measures ANOVA	Visual = 0.95 ± 0.07 vs Visual and auditory = 1.17 ± 0.06 Average calcium activity (ΔF_1_/F_0_/s) following Log_10_ transformation	*p* < 0.001	55 Increasing Neurons
4l	For non-increasing multimodal neurons Average response to visual stimulus vs visual and auditory stimulus	One-Way Repeated measures ANOVA	Visual = 0.71 ± 0.11 vs Visual and auditory = 0.77 ± 0.11 Average calcium activity (ΔF_1_/F_0_/s) following Log_10_ transformation	*p* = 0.545	38 Non-increasing Neurons
4m	For multimodal neurons percentage change in visual response with sound for cells with Low vs Medium vs High Co-activity (% of trials)	One-Way ANOVA	Low (<25 %) = −19 ± 16 %, Medium (25–50 %) = + 22 ± 12 % High (>50%) = + 49 ± 9 % Percentage change in calcium activity (ΔF_1_/F_0_/s)	Low vs High *p* = 0.002	100 Neurons
4n	Correlation between sound-driven enhancement and later tone response plasticity for the paired tone	Spearman’s Rank Correlation	Sound-driven enhancement during coincident presentation vs later increases in tone response after Audio-visual pairing (*r* = 0.89)	*p* < 0.001	94 Neurons
4o	Correlation between sound-driven enhancement and later tone response plasticity for unpaired tone	Spearman’s Rank Correlation	Sound driven enhancement during coincident presentation vs unpaired tone response (*r* = −0.25)	*p* = 0.545	94 Neurons
4p	For multimodal neurons change in association (r) between cell pairs of Low vs Medium vs High co-activity (% of trials)	One-Way ANOVA	Low (<25 %) = −0.003 ± 0.014, Medium (25–50 %) = 0.043 ± 0.016 High (>50%) = 0.070 ± 0.024 Change in association (r)	Low vs High *p* = 0.028	2084 cell pairs

The data for Fig. 4 uses 332 neurons taken from six cortical regions across six animals

### Co-activity during pairing correlates with plasticity

Sound can both enhance and suppress visually driven responses in V1, but the network consequences of this modulation are unclear^7^. Our simulation predicted that bouts of coincident neural activity were triggered by sound-driven enhancement of visual activity and this drove both network strengthening and tone plasticity after pairing (Fig. 4e–k). We used our calcium imaging data to probe this prediction and link activity during audio-visual pairing trials to subsequent plasticity measured after pairing. Consistent with the predictions of our simulation, we found tone plasticity after audio-visual pairing was greatest at multimodal neurons that showed sound-driven enhancement of visual activity during pairing (Fig. 4l). Sound driven enhancement increased the co-activity between pairs of multimodal neurons during pairing trials (Fig. 4m) and positively correlated with subsequent plasticity to the paired (Fig. 4n), but not the unpaired tone (Fig. 4o). In keeping with the predictions of our simulation (Fig. 4k), we found that co-activity between multimodal cell pairs was positively correlated with the degree of functional network strengthening shown after pairing (Fig. 4p). The results of our simulation in combination with our experimental data suggest that sound-driven enhancement of visual activity may act to strengthen functional multimodal assemblies during audio-visual experience by driving bouts of co-activity.

## Discussion

Here we asked how inputs from two senses (auditory and visual) interact in neural-circuits of V1, and whether epochs of coincident sensory activity can drive neural plasticity or adaptation. We addressed these questions using a combination of GEVI and GECI imaging and found that coincident audio-visual pairing can modify neural-circuit properties. A subset of multimodal neurons exhibited an enhanced response to a tone that had been repeatedly and simultaneously presented with a visual stimulus. These multimodal neurons were driven by the paired visual stimulus and shared small subnetworks. After coincident audio-visual pairing, multimodal neurons with increased tone responses became more associated with each other and less associated with other local neurons. Strengthened network associations occurred during bouts of co-activity resulting from sound-driven response enhancement. Our work suggests that the primary sensory cortex can integrate coincident streams of multisensory activity by strengthening functional multimodal assemblies to leave a trace of multisensory experience in the cortex.

We found that sensory driven plasticity is focused at a subset of multimodal neurons. Previous work has shown adaptive plasticity in V1 to presentation of paired visual stimuli^31^, spatial sequences^32^ and naturalistic movies^33^. All these approaches induce neural adaptation over timescales similar to those we observed in our experiments. Sensory driven plasticity can be rapid and short lasting such as that seen during contrast adaptation^34^, or occur over minutes and be expressed for hours, such as that seen during receptive field expansion^35^. The plasticity we report here develops over repeated trials across approximately 45–60 min. Previous work has shown a positive correlation between stimulus reinforcement and the persistence of a modified response^36^. We found pairing driven plasticity was maintained over a 24 h period and resulted in the sharpening of auditory tuning curves in a subset of multimodal neurons. Interestingly, the population of multimodal neurons that developed an auditory response (having shown no response in the baseline) did not have sharper tuning after pairing (Supplementary Fig. 1j). Further work is required to assess the persistence of cross-modal plasticity to see if emerging auditory responses can re-tune response selectivity and the degree to which emerging responses contribute to the population response. Such work is likely to involve chronic preparations and repeated training epochs delivered over many days^37–41^.

In our study we did not observe any strengthening of cortical responses following repeated presentation of unpaired visual or auditory stimuli. In fact, we observed a small but significant reduction in the response of multimodal neurons to repeated tone presentation. The reduction in activity we observed following repeated unpaired tone presentation may represent a form of adaptation similar to that reported following repeated presentation of visual stimuli in V1^42,43^. Some studies of cortical adaption have shown stimulus-driven plasticity can develop over days in both V1^38,40^ and primary auditory cortex (A1)^44^. Further work is required to understand how adaptive responses following repeated stimulus presentation may lead to response potentiation and ultimately perceptual learning^45^.

We found a subset of multimodal neurons had enhanced tone responses after audio-visual pairing. These neurons exhibited sound-driven enhancement of visual activity and were responsive to the paired visual stimulus. Although the modulation of visual responses by sound has been described before, its role in multisensory integration is unclear^4,5,7^. Our network simulation and experimental data suggest that sound-driven response enhancement increases coincident activity between neurons during audio-visual pairing trials, leading to neural-circuit modifications, possibly through Hebbian-like synaptic plasticity mechanisms. In our dataset, multimodal neurons were functionally associated and may therefore share synaptic connections^23,46^. Thus, coincident activity during audio-visual pairing may drive synaptic strengthening. Indeed, the degree of co-activity between pairs of multimodal neurons during audio-visual pairing trials did predict later changes in functional network associations. However, we cannot exclude the influence of other factors such as neuromodulation, common inputs outside of L2/3 or changes in inhibition, all of which are known to modulate activity in sensory cortex^47,48^. Furthermore, the auditory and visual tuning properties of multimodal neurons were not correlated in our experiments suggesting that multiple layered network representations may work in concert to maximise the flexibility of cortical responses^49^.

The selective strengthening and weakening of functional neural assemblies has been reported in motor cortex during learning^28^ and may improve the decoding capabilities of the cortex^50,51^. Such plasticity re-emphasises the important role that cortical subnetworks play in both sensory processing^52^ and sensory experience dependent network plasticity^22,30^. Interestingly, the observed changes in functional network associations were evident after audio-visual pairing during periods of spontaneous activity. The spontaneous reactivation of those neural-circuits involved in sensory processing is thought to contribute to memory consolidation and has been shown to occur in the hippocampus and neocortex^36,53^. Thus, one consequence of the functional network plasticity we observe may be to promote reactivation of the circuits involved in the processing of audio-visual stimuli during spontaneous activity.

We found cross-modal feature selectivity at multimodal neurons, our findings are supported by functional studies in both cat and rat cortex^3,8–10^. However, one possibility is that these responses are attributable to attentional enhancement, which is known to modulate visual cortical activity^54^. We think this unlikely, as a subset of neurons consistently responded to specific individual tones on multiple trials. Furthermore, others have shown no changes in pupil size (proxy for attentional enhancement) during audio-visual pairing using stimuli similar to those reported here^7^. However, we can not discount the possibility that our classification of multimodal neurons may be contaminated by signals relating to attentional enhancement.

Our experimental data and network simulations suggest that sound-driven enhancement of visual activity can drive network level plasticity in V1. A number of possible neural-circuit mechanisms are thought to be recruited by sound in order to modulate visual activity in V1. Many of these circuits involve complex inhibitory interactions. Multiple inhibitory neuron subtypes such as those expressing, parvalbumin (PV), somatostatin (SOM) and vasoactive intestinal peptide (VIP) are known to exhibit heterogeneous responses to sound presentation in V1^4^. VIP expressing interneurons show a strong suppression of activity during sound presentation which may modulate the response of excitatory neurons to visual stimuli^4^. VIP interneurons predominantly inhibit SOM interneurons, which form inhibitory connections with PV expressing interneurons^55^ and distal dendrites of L2/3 pyramidal neurons^56^. Suppression of VIP interneurons during sound presentation may lead to either sound suppression (disinhibition of SOM leading to greater inhibition of pyramidal dendrites) or sound enhancement (disinhibition of SOM leading to greater inhibition of PV cells and reduced inhibitory drive to excitatory cells). The picture is made more complicated by recent findings showing response suppression and enhancement in V1 are dependent on certain temporal features of the presented sensory stimuli^7^. In our work, voltage imaging experiments found that audio-visual pairing was accompanied by a shift from hyperpolarising to depolarising cortical activity. Our voltage imaging results must be interpreted with care as a shift from hyperpolarising to depolarising activity may occur in several ways including reductions in inhibition, increases in excitatory activity or a combination of both. Changes in inhibitory activity are known to be permissive for synaptic plasticity and it is possible that both scenarios may work in concert^57^. Further work is required to understand the way in which cross-modal modulation may gate plasticity in primary sensory cortices, and particularly the role of inhibitory sub-types in this modulation. Finally, further work is required to establish if the network plasticity observed here is a general mechanism adopted by multimodal neurons to integrate visual information with the plethora of other non-visual signals that have been reported in mouse V1^18,58–67^.

## Methods

### Animals and surgery

Experiments were conducted according to the United Kingdom Animals (Scientific Procedures) Act 1986. For lightly anaesthetised calcium imaging experiments, we used six adult (P60–120) mice expressing the GECI GCaMP6f in L2/3 excitatory neurons under inducible Cre and tTA activities: *Rasgrf*-2A-dCre-tTA-GCaMP6f. For awake behaving imaging, we used four adult (P60–120) mice and injected AAV2/1 EF1a CyRFP-GSG-P2A-GCaMP6s-WPRE into V1 during implantation of the cranial window. For voltage imaging experiments under light anaesthesia, we used five adult (P60–120) mice expressing the GEVI Chi-VSFPBfly1.2 under the *Camk2A*-tTA promotor: *CaMk2A*-tTA-Chi-VSFPBfly1.2. Cranial windows were implanted over the monocular primary visual cortex in ketamine-xylazine anaesthetised mice (ketamine 0.15 mg/g and xylazine 0.015 mg/g of body weight)^22,68^. After surgery animals were transferred to the 2-P imaging set-up and allowed to recover until light whisking was observed. Anaesthetic levels were maintained and monitored with a low dose of ketamine/xylazine as previously described^69^. Body temperature was monitored with a probe and maintained at 37 °C by a heating pad (Harvard Apparatus). Artificial tears were regularly applied to prevent the eyes from drying (Viscotears Liquid Gel, Alcon Novartis). For longitudinal awake behaving imaging experiments, all mice recovered for 2–3 weeks after surgery prior to imaging which was conducted during a baseline period and then again 24 h later.

### Functional 2-P imaging

For functional 2-P voltage imaging, we scanned a 64 × 64 pixel frame (Ultima IV, Bruker, Coventry UK) at 22 Hz using a Ti-Saphire Laser (Chameleon Coherent, Ely, UK) set at 920 nm with a ×16 Nikon objective (NA, 0.8). The average laser power was set to <50 mW. Emission light was separated from excitation light by a dichroic mirror (Multiphoton-LP-Beamsplitter 720 DCXXR, Chroma, Bellows Falls, VT) and filtered with an infrared blocking filter ET700sp-2p8 (Chroma). mCitrine and mKate2 emission separation was achieved using a FF580-FDi01 25 × 36 (Semrock, Rochester, NY) dichroic mirror with mCtrine being filtered by a FF01–542/50–25 filter (Semrock) and mKate2 by a BLP01–594R-25 filter (Semrock). During 2-P calcium imaging, we scanned a 128 × 128 pixel frame at 10 Hz, otherwise all settings were the same. GCaMP6f emission was separated by a BA460–510HQ filter (Olympus).

### Sensory stimuli

Auditory sensory stimulation consisted of pseudo-random presentation of pure tones (1–8 kHz, 4 s duration, 70 dB intensity) presented bilaterally from speakers placed at a distance of 10 cm from the mouse. Visual sensory stimulation consisted of drifting gratings in eight directions (0°–360°, in 45° steps), presented in a pseudo-random order, with a spatial frequency of 0.04 cycles/degree and a temporal frequency of 2 Hz at 100 % contrast. Visual and auditory stimuli and specific paired or unpaired stimulus sets were presented for 4 s followed by a 2 s interval. During the mapping and re-testing phases we took an average of five trials of each stimulus before and five trials of each stimulus after pairing. Each trial started with a 12 s period without stimulation, so that the total trial duration was 1 min. Trials of spontaneous data were collected for 5–10 min without either visual or auditory stimulation in the dark, before and after pairing. For pairing trials, we paired a randomly selected visual and auditory tone over 15–100 trials. The paired stimuli were always presented simultaneously, however, in a subset of experiments we investigated the issue of temporal pairing by measuring changes in the response to a tone presented immediately after a grating (Supplementary Fig. 1l–right). We found no clear strengthening response, but instead adaption-like responses similar to those seen following repeated presentation of a single modal stimulus (Fig. 1i, m). Pairing trials were randomly interleaved with unpaired stimulation trials using an auditory tone that was at least ± 3 kHz from the randomly selected paired tone and a visual stimulus that was orthogonal to the paired visual stimulus. In a subset of experiments, awake behaving animals were presented with a novel drifting grating and tone pairing stimulus set to examine the extent to which our findings generalised to different visual stimuli (Supplementary Fig. 1k). Exposure to the novel stimulus set resulted in levels of plasticity that were comparable to our original visual stimuli set (Fig. 1l and Supplementary Fig. 1k). The persistence of tone plasticity was measured across imaging sessions using the same stimuli as had been presented on the baseline testing day. To further test the tenacity of the pairing driven plasticity we conducted a subset of experiments where paired tone strengthening was first induced and then the paired tone was repeatedly (30–40 trials) presented in isolation (Supplementary Fig. 1l–left).

### Calcium imaging analysis

Calcium imaging data were analysed using previously published methods^22,70–72^. Data were full frame registered using the motion correction software ‘Moco’^73^. Neurons were selected based on the mean normalised maximum intensity projection of the data by hand and the nucleus was excluded from selection. Mean-normalised maximum projections were calculated by normalising the maximum projection, calculated on a running average of 20 frames, by the mean projection. Fluorescence traces were calculated as the average fluorescence of pixels lying within the cell in each frame. To remove slow changes in raw fluorescence traces, the 10th percentile value of the fluorescence distribution in a ±8 s window was subtracted from the raw fluorescence signal. ΔF_1_/F_0_ signals were calculated by dividing the raw fluorescence signal by the median of each cell’s fluorescence distribution. To calculate cellular activity, we smoothed each trace and generated an average from five trials. We then took the activity above a 10%ΔF_1_/F_0_ threshold and measured the integral of the average ΔF_1_/F_0_ signal. Orientation selectivity was calculated as previously described^22,23^. To measure the degree of auditory tuning to tones of different frequencies we adapted a previously published method^16^. For each neuron, we normalised responses to the best tone response and then fitted a line to the normalised tuning curves, the slope *m* of the linear fit through the data was used as an index of tuning sharpness (Supplementary Fig. 1a, j**)**.

### Voltage imaging analysis

Voltage signals measured with GEVIs are dominated by the neuropil signal, rather than single cell activity. To analyse voltage imaging data, we adapted previously published methods^19,20,74^. All 2-P data underwent mean 3D filtering with a 2 × 2 × 2 pixel resolution. Absolute mCitrine (donor) and mKate2 (acceptor) response sequences (ΔF/F) were obtained by subtraction of the average pre-stimulus baseline from the frames encompassing both the baseline and the event. Ratio images were then obtained by dividing the mKate2 ΔF/F sequence by the mCitrine ΔF/F sequence. Each region was split into a 3 × 3 grid (40 × 40 µm squares) and the average activity over each region across trials was measured. Average traces (five trials) were then used to estimate voltage activity by taking the integral of the average ratio trace.

### Definition of multimodal neurons

Neurons were considered to be multimodal if they exhibited an average calcium response that was greater than a threshold of 15%ΔF_1_/F_0_ in both the visual and auditory stimulation conditions and if the averaged response exhibited a clear time-locked onset to the presentation of the stimulus as determined by visual inspection (blind to the condition). The same parameters were used to define neurons responsive to auditory and visual stimuli only, as well as neurons that were non-responsive in all conditions. The total activity of multimodal neurons during either visual or auditory stimulation was found to be similar by summing all activity during presentation in each condition and normalising to total visual activity (Supplementary Fig. 1d inset). Where indicated in the text, we sub-divided neurons into three categories based on their response to the paired auditory and visual stimuli when presented independently during the baseline mapping phase: (1) responsive to both the paired auditory and visual stimuli, (2) responsive to the paired visual stimuli only and (3) responsive to the paired auditory stimuli only. Neurons were classified as responding if they showed an average response to presentation of the stimuli even if this was not their maximal response. Neurons that did not respond to either the paired visual or paired auditory stimuli were excluded from this analysis. Neurons were defined as exhibiting an increased response to tone presentation following audio-visual pairing if their average response was 20% greater than their baseline response to the paired tone.

### Network correlation analysis

Network correlation analysis was performed as described previously^22^. Briefly, periods of spontaneous cortical activity were estimated by acquiring calcium activity in the dark without either visual or auditory stimulation. These data were used to calculate pairwise correlation coefficients between calcium transients of active neurons in the same cortical region. The average or summed correlation strength was taken from positive correlations. Epochs of spontaneous data were collected both before and after audio-visual pairing. For analysis of functional associations before and after audio-visual pairing, we calculated the percentage correlation coefficient attributable to neurons that either exhibited an increased tone response, or did not, as a fraction of all summed correlation coefficients for each multimodal neuron (Fig. 3). Similar analysis was conducted for awake animals using periods of auditory and visual sensory stimulation before and after pairing (Supplementary Fig. 3c, d).

### Computational network modelling

Network simulations were performed with the ‘NumPy’ Python package (Supplementary Table 4). For our analysis, we simulated 50 independently generated networks of 200 neurons each and pooled the results of the stimulus pairing protocol across all networks. We used a simple firing rate neuron model, given by the transfer function *g*(*x*) defined below^75,76^.

1 $$\begin{array}{*{20}{c}} {g(x)} =\, {0\,\qquad{\mathrm{if}}\quad x \, < \, 0}\hfill \\ \qquad=\, {(r_{{\mathrm{max}}} - r_0){\mathrm{tanh}}(x/(r_{{\mathrm{max}}} - r_0))\qquad{\mathrm{if}}\quad x \ge 0} \end{array}$$

This leads to firing rates with a baseline of *r*_0_ and a maximum of *r*_max_. Following Rajan et al.^76^, the firing rates *y*_*i*_ of neuron *i* driven by external input *H*_*i*_ in a network are described as follows:

2 $$\frac{{dy_i}}{{dt}} = - y_i + \mathop {\sum}\limits_{j = i}^N {W_{ji}g(y_j) + H_i}$$

where *W*_*ji*_ is the weight of the synaptic connection from neuron *j* to neuron *i*.

Our network consisted of 200 excitatory neurons and 40 inhibitory neurons. The dynamics of both inhibitory (I) and excitatory (E) neurons are described by Eqs. (1), (2). The network had dense all-to-all synaptic connectivity in the E-E, E-I and I-E populations, and no I-I connectivity. Self-connections, or autapses, were not permitted. As such, *W*_*ij*_ in Eq. (2) takes the form of a square matrix with size 240^2^. The strength of the inhibitory synapses was initially set so that inhibitory currents roughly balanced excitatory currents in the network. We used the BCM learning rule to model excitatory synaptic plasticity of recurrent excitatory to excitatory (E-E) and excitatory to inhibitory (E-I) synapses^29^.

3 $$\frac{{dW_{ij}}}{{dt}} = \alpha y_iy_j(y_j - \theta _j)$$

4 $$\frac{{d\theta _i}}{{dt}} = \tau _\theta \left( {\frac{{y_{i^2}}}{{y_0}} - \theta _i} \right)$$

where *α* is the learning rate, *θ*_*i*_ refers to the sliding threshold which determines whether potentiation or depression occurs for synapses onto each neuron *i*, and which depends on the neuron’s recent postsynaptic activity, *y*_*i*_. *τ*_*θ*_ is the rate at which *θ*_*i*_ is modified to maintain the postsynaptic firing rate at its homeostatic target, *y*_0_. The BCM learning rule has both a Hebbian component and a homeostatic component. We use a homeostatic rule to model inhibitory synaptic plasticity of recurrent inhibitory to excitatory (I-E) weights^77^,

5 $$\frac{{dW_{\,\,\,\,\,\,\,ij}^{IE}}}{{dt}} = \eta y_i(y_j - y_0),$$

where *y*_0_ is the homeostatic target firing rate, *η* is the learning rate, and $W_{\,\,\,\,\,\,\,ji}^{IE}$ is the weight of the synaptic connection from inhibitory neuron *i* to excitatory neuron *j*. Excitatory weights are bounded so that their values lie between 0 and *w*_max_, and inhibitory weights are bounded so that they lie between −*w*_max-inh_ and 0. The homeostatic target, *y*_0_, is the same for both inhibitory plasticity and the homeostatic component of BCM plasticity.

We simulated three classes of neurons, defined by the feedforward inputs they receive: visual neurons, auditory neurons and multimodal neurons. Each visual or auditory neuron was randomly assigned a preferred visual or auditory stimulus. Multimodal neurons were independently assigned both a preferred visual and auditory stimulus. Of the 200 excitatory neurons in our network, 80 were visual, 80 were multimodal and 40 were auditory. In order to ensure that a sufficient number of neurons belonged to each multimodal stimulus combination, we used four visual stimuli (V_1_, V_2_, V_3_, V_4_) and two auditory stimuli (A_1_, A_2_). We focused on multimodal neurons that preferred the paired or unpaired stimuli (V_1_ or V_2_ and A_1_ or A_2_); the pattern of connectivity after development was identical for multimodal neurons with the remaining stimulus preferences (V_3_ or V_4_ and A_1_ or A_2_). We measured co-activity during pairing trials for each pair of neurons as the average Pearson correlation coefficient of both neurons firing rate whenever the paired grating and tone were presented to the network. The external input current *H*_*i*_ for each neuron *i* is given by

6 $$H_i = H_0 + H_{{\mathrm{vis}}}\delta ({\mathrm{vis}}) + H_{{\mathrm{aud}}} \, \delta \, ({\mathrm{aud}}),$$

Where *H*_0_ is a constant, and *δ*(vis) is 1 if the preferred visual stimulus is present and 0 otherwise. Likewise, *δ*(aud) is 1 if the preferred auditory stimulus is present and 0 otherwise. Excitatory weights were initially uniform, set at $\frac{{w_{{\mathrm{max}}}}}{2}$. We simulated development by letting synaptic weights evolve under Eqs. (3)–(5), as we presented randomly chosen visual and auditory stimuli simultaneously to the network. We changed the identity of the visual and auditory stimuli every 500 ms, and development is simulated for 5000 s. Throughout the development, we used *H*_vis_ = 10 and *H*_aud_ = 7. When simulating the plasticity protocol, one visual and one auditory stimulus were paired, and one visual and one auditory stimulus were unpaired. Throughout the stimulus pairing protocol, we used *H*_vis_ = *H*_aud_ = 10. In addition to the external inputs given by Eq. (6), throughout the pairing protocol we added independent Ornstein-Uhlenbeck noise to each excitatory neuron, with *σ* = 2.5, *μ* = 0, *τ* = 50 ms^78^. We randomly interleaved paired or unpaired trials, each 500 ms long and with 50% probability. During paired trials V_1_ and A_1_ were presented simultaneously. During unpaired trials either V_2_ or A_2_ are presented to the network, each with 50% probability. Between each trial we allowed the network activity to reset by running the network without any visual or auditory stimuli for 2500 ms. Throughout the pairing protocol, inhibitory weights were fixed, and a uniform, static threshold of potentiation for each neuron: *θ*_*i*_ = 9 for all *i*. This is under the assumption that these slow homeostatic forms of plasticity do not have a substantive effect during the pairing protocol, while they are required for the development of selectivity prior to the pairing protocol. We also increase *α* to 5 × 10^−5^ Hz during the pairing protocol.

### Sound driven enhancement and co-activity analysis

To determine the degree of co-activity between neurons during pairing trials, we measured the percentage of trials during which cell pairs were both active above a 15%ΔF_1_/F_0_ threshold. Neurons were then split into low (<25%), medium (25–50%) or high (>50%) co-activity groups. Sound driven enhancement was estimated by looking at the change in activity during simultaneous presentation of the paired visual and auditory stimulus as a percentage of the summed activity during separate presentation.

### Statistics

All statistical analysis was performed either in Matlab or SigmaPlot v.13 (Systat Software Inc.). Comparisons were made using one- or two-sided, parametric or non-parametric statistics and correction for multiple testing was conducted using either the Holm-Šidák or Dunn’s method as appropriate. Correlations were run using the Pearson’s or Spearman’s Rank correlation tests. Tests of proportions were conducted with Chi-Squared tests or the z-test of proportions.

### Reporting summary

Further information on research design is available in the Nature Research Reporting Summary linked to this article.

## Supplementary information

Supplementary Information

Reporting Summary

## Data Availability

The data and code that support the findings of this study are available from the corresponding author upon reasonable request. Source data underlying Figs. 1–4 are available as a Source data file.

## Source Data

Source Data

## Code availability

The code for running the network simulations is available at https://github.com/yannaodh/multimodal_2019.
