# Supplementary material for: Audio-visual experience strengthens multisensory assemblies in adult mouse visual cortex
Source: Nat Commun. 2019 Dec 12;10:5684. doi: 10.1038/s41467-019-13607-2 (PMC6908602; doi:10.1038/s41467-019-13607-2)
Supplement: Supplementary file 1 — Supplementary Information [file 41467_2019_13607_MOESM1_ESM.pdf]

Supplementary Information

Audio-visual experience strengthens multisensory assemblies in adult mouse visual cortex

Thomas Knöpfel[1], Yann Sweeney[3], Carola I. Radulescu[2,4], Nawal Zabouri[2,4], Nazanin Doostdar[2,4], Claudia Clopath[3] and Samuel J. Barnes[2,4] *

1. Laboratory for Neuronal Circuit Dynamics, Department of Brain Sciences, Imperial College London, W12 0NN, UK
2. Department of Brain Sciences, Division of Neuroscience, Imperial College London, Hammersmith Hospital Campus, Du Cane Road, London, W12 0NN, UK.
3. Department of Biomedical Engineering, Imperial College London, South Kensington Campus, London, SW7 2AZ, UK.
4. UK Dementia Research Institute at Imperial College London, UK.

*Corresponding author Samuel J. Barnes[2,4] samuel.barnes@imperial.ac.uk

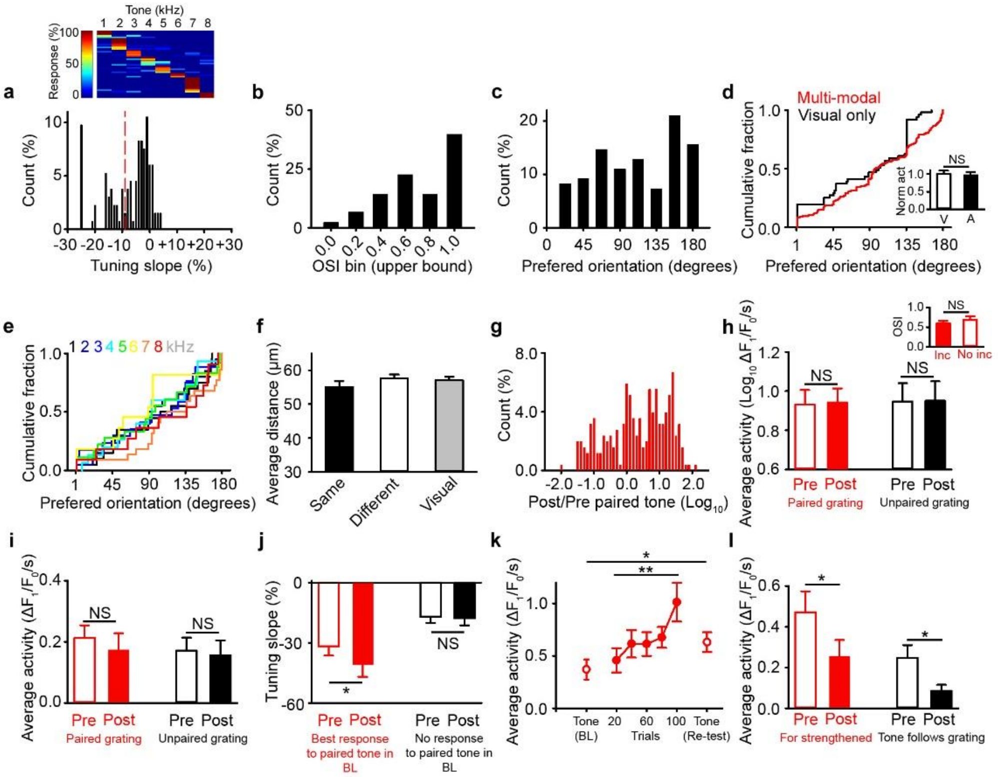

Supplementary Figure 1

Supplementary Figure 1: Multimodal neurons show auditory and visual feature selectivity

a) Top: Heatmap from the neurons that are left of the red dashed line: i.e. those that are most strongly feature selective in the histogram below. The heatmap is sorted first by best tone response and then by auditory tuning slope using a previously published method [16]. Bottom: Histogram of auditory tuning slopes for multimodal neurons. Red dashed line indicates those neurons with a tuning slope giving a > -10 % change per 1 kHz change. **b)** Histogram of orientation selectivity index (OSI) for multimodal neurons. **c)** Histogram of preferred orientation for multimodal neurons. **d)** Cumulative fraction showing distribution of orientation preference for visual only neurons (black) and multimodal neurons (red). Inset: Summed visual (open) and auditory (filled) activity at multimodal neurons, values are normalized to visual activity. **e)** Cumulative fraction plots showing preferred visual orientation for multimodal neurons that respond best to tones of different frequencies as indicated by colour code. **f)** No difference between the average distance between neurons with the same best tone response (black, filled) and neurons with different tone responses (black, open) or neurons that do not respond to tones but instead respond to visual stimuli (grey, filled). **g)** Histogram of plasticity scores (Log_{10}) for multimodal neurons with change in mean calcium activity ($\Delta F_1/F_0$/s) (after pairing) to the paired tone normalized to baseline mean calcium activity to the paired tone from anaesthetised and awake animals. **h)** Average calcium activity ($\Delta F_1/F_0$/s) following Log_{10} transformation, for all multimodal neurons in response to either the paired grating (red) or the unpaired grating (black) before (open) or after (filled) paired and unpaired trials in lightly anaesthetised animals. Inset: Average orientation selectivity index for multimodal neurons that either show increased activity (filled) or do not (open) after pairing. **i)** Average calcium activity ($\Delta F_1/F_0$/s) following Log_{10} transformation, for all multimodal neurons in response to either the paired grating (red) or the unpaired grating (black) before (open) or after (filled) paired and unpaired trials in awake animals. **j)** Auditory tuning slopes for multimodal neurons measured in awake animals for neurons with a best response to the paired tone (red) in the baseline before (open) or after (filled) pairing or neurons that did not have a response to the paired tone

(black) in the baseline before (open) and after (filled) pairing. **k)** Average calcium activity ($\Delta F_1/F_0$/s) response of multimodal neurons to audio-visual pairing of a novel grating and tone pairing set in awake animals. Open circles are tone presentation in the baseline and re-testing in the baseline, calcium response to pairing stimuli is shown as filled circles. **l)** Red bars show persistence of strengthened paired stimulus response at multimodal neurons in awake animals before (red open) and following repeated (30 – 40 trials) single modality tone presentation (red, filled). Black bars show response of multimodal neurons to a tone in the baseline (black, open) and following repeated presentation immediately after a visual stimulus (black, filled) (30 – 40 trials). Supplementary Figure 1 a-h uses recordings from 332 neurons taken from six cortical regions across six animals in light anaesthetic conditions and Supplementary Figure 1 g, i-l uses 408 neurons from four cortical regions across four animals in awake conditions. In all panels, *p<0.05, **p<0.01 (see Supplementary Table 1). Error bars: mean and ± S.E.M. Source data are provided as a Source Data file.

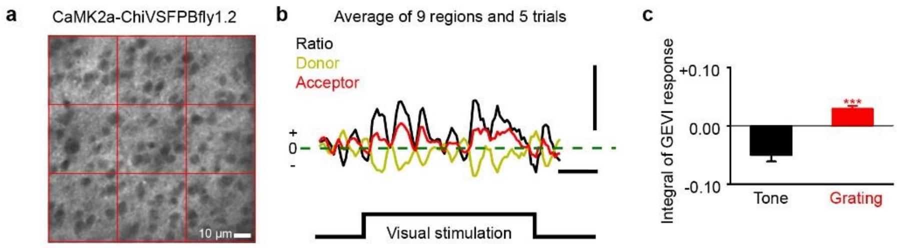

Supplementary Figure 2

Supplementary Figure 2: Spatio-temporal averaging of GEVI

a) Example region taken from L2/3 of V1 in a CaMK2A-Chi-VSFPBfly1.2 expressing adult mouse under anaesthetised conditions showing grid used to calculate spatio-temporal average (red, lines). Scale bar: 10 µm. **b)** Example response trace from the average of five trials across nine cortical regions to the same visual stimulus showing the response of the donor (mCtrine: yellow-green trace), acceptor (mKate2: red trace) and the ratio (black trace calculated as: Ratio = Acceptor: mKate2 / Donor: mCitrine. Scale bars are 1 % and 1 s. **c)** Integral of GEVI response to auditory tone (black) and visual drifting grating stimulation (red). Supplementary Figure 2a-c uses 358 regions taken from five animals under anaesthetised conditions. For all panels, ***p<0.001 (see Supplementary Table 2). Error bars: mean and ± S.E.M. Source data are provided as a Source Data file.

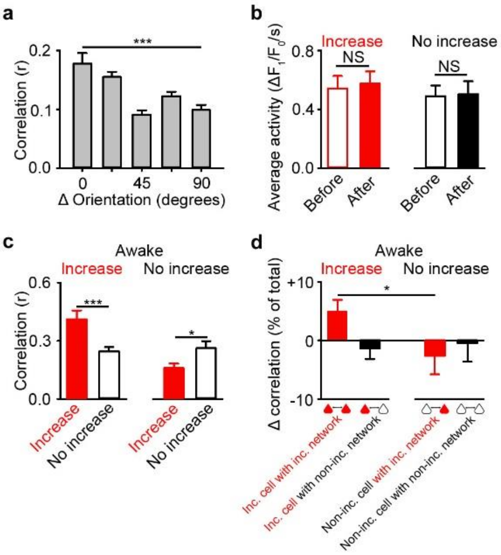

Supplementary Figure 3

Supplementary Figure 3: Spontaneous activity levels are similar before and after pairing for both increasing and non-increasing neurons

a) Visual feature selective subnetworks comprising multimodal neurons are evident during periods of spontaneous activity. **b)** Plot of spontaneous calcium activity ($\Delta F_1/F_0/s$) before (open) and after (filled) audio-visual pairing for neurons that have an increased response (red) to the tone following audio-visual pairing and neurons that do not (black). **c)** Data from awake animals during sensory stimulation showing correlation strength (r) prior to audio-visual pairing between multimodal neurons that either: have an increased response to the paired tone after audio visual pairing (left) or do not (right). Correlation value in each case is with other neurons that go on to either: have greater tone responses (red, filled) or do not (black, open) following audio-visual pairing trials. **d)** Data from awake animals during sensory stimulation showing change in correlation between increasing neurons and increasing network/non-increasing network (left) and non-increasing neurons and increasing/non-increasing networks (right). The data for Supplementary Figure 3a-b uses 93 multimodal neurons taken from five cortical regions across five animals in anaesthetised conditions and Supplementary Figure 3 c-d uses 160 neurons from four cortical regions across four animals in awake conditions. In all panels, *p<0.05, ***p<0.001 (see Supplementary Table 3). Error bars: mean and ± S.E.M. Source data are provided as a Source Data file.

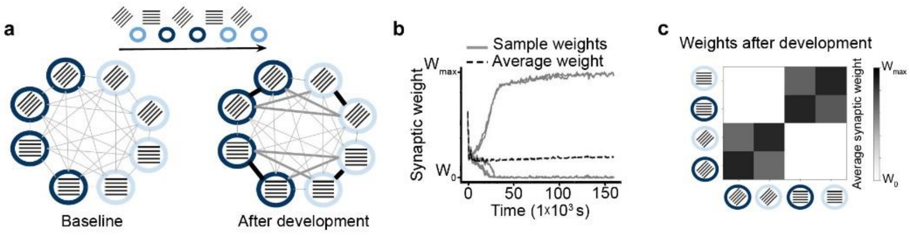

Supplementary Figure 4

Supplementary Figure 4: Development of multimodal network model

a) Schematic illustrating the development of the reciprocal network model where repeated presentation of different auditory (light and dark blue circles) and visual (grating icons) stimuli modify synaptic weights. **b)** Example of synaptic weight evolution during the development phase of the network simulation showing average synaptic weight (dashed line) and sample weights that are either strong or weak (grey lines). **c)** Weight matrix for different combinations of multimodal neurons, showing weight strengths after development. (see Supplementary Table 4).

Statistical Comparisons For Supplementary Figure 1 d – l			Centre and dispersion or fit	Result	
Panel	Comparison	Test		p value	N value
S1d	Visual Neurons vs Multimodal neurons Preferred orientation (degrees)	MWRST	Visual = 101, 34 – 136°<br><br>Multimodal = 97, 53–148°,	p = 0.064	Visual = 51 neurons<br><br>Multimodal = 133 neurons
S1d inset	Visual activity Vs Auditory activity	t-test	Visual normalized activity = 1.00 ± 0.09<br>vs<br>Auditory normalized activity = 0.963 ± 0.09	p = 0.768	N = 133 neurons
S1e	Preferred orientation for each best frequency of multimodal neurons	One-Way ANOVA	1 kHz = 102 ± 13°<br>2 kHz = 95 ± 12°<br>3 kHz = 96 ± 15°<br>4 kHz = 94 ± 14°<br>5 kHz = 95 ± 14°<br>6 kHz = 81 ± 18°<br>7 kHz = 117 ± 12°<br>8 kHz = 110 ± 18°	p = 0.790	N = 133 neurons
S1f	Distance to neuron with the same best tone vs distance to neuron with a different best tone	One-Way ANOVA	Same = 55 ± 2 µm<br>vs<br>Different = 58 ± 1 µm<br>vs<br>Visual only= 57 ± 1 µm	p = 0.384	N = 475 distance measures
S1g	Distribution of plasticity scores for multimodal neurons normalised to baseline paired tone activity (awake and anaesthetised combined)	Descriptive statistics	Normalized change in response ($\Delta F_1/F_0$/s Log_{10}) = 0.30 ± 0.11	NA	N = 296 neurons
S1h	Average activity of multimodal neurons in response to the paired grating Before vs After Pairing In lightly anaesthetised animals	One-Way Repeated Measures ANOVA	Pre-pairing = 0.93 ± 0.07<br>vs<br>Post-pairing = 0.94 ± 0.07<br><br>Average activity($\Delta F_1/F_0$/s) following Log_{10} transform	p = 0.901	N = 91 neurons
S1h	Average activity of multimodal neurons in response to the unpaired grating Before vs After Pairing In lightly anaesthetised animals	One-Way Repeated Measures ANOVA	Before = 0.95 ± 0.09<br>vs<br>After = 0.95 ± 0.10<br><br>Average activity ($\Delta F_1/F_0$/s) following Log_{10} transform	p = 0.984	N = 46 neurons
S1h Inset	OSI for multimodal neurons that: exhibited an increased tone response Vs those that do not after pairing	t-test	Increased response (OSI = 0.59 ± 0.04)<br>Vs<br>No increased response (OSI = 0.69 ± 0.05)	p = 0.098	N = 108 neurons

S1i	Average activity of multimodal neurons in response to the paired grating Before vs After Pairing In awake animals	t-test	Pre-pairing = 0.21 ± 0.04 vs Post-pairing = 0.17 ± 0.05 (Average activity $\Delta F_1/F_0$/s)	p = 0.559	N = 160 neurons
S1i	Average activity of multimodal neurons in response to the unpaired grating Before vs After presentation In awake animals	t-test	Before = 0.17 ± 0.04 vs After = 0.16 ± 0.05 (Average activity $\Delta F_1/F_0$/s)	p = 0.812	
S1j	Average change in auditory tuning of multimodal neurons showing response to paired tone in the baseline and strengthening after pairing In awake animals	Paired t-test	Pre = -31.7 ± 4.6 vs Post = -40.8 ± 6.0 (Tuning slope %)	p = 0.010	N = 36 neurons
S1j	Average change in auditory tuning of multimodal neurons not showing a response to paired tone in the baseline and strengthening after pairing In awake animals	Paired t-test	Pre= -17.0 ± 3.0 vs Post = -17.6 ± 3.7 (Tuning slope %)	p = 0.772	N = 68 neurons
S1k	Change in response of multimodal neurons to paired tone before and after pairing in awake animals	t-test	Tone BL = 0.37 ± 0.10 Tone re-test = 0.63 ± 0.09 (Average calcium activity $\Delta F_1/F_0$/s)	p = 0.046	N = 160 neurons
S1k	Change in response of multimodal neurons to repeated audio-visual presentation in awake animals	Paired t-test	Pairing trials (15-20) = 0.46 ± 0.12 Pairing trials (95-100) = 1.01 ± 0.18 (Average calcium activity $\Delta F_1/F_0$/s)	p = 0.002	N = 160 neurons
S1l	Average activity of multimodal neurons strengthened by pairing in response to repeated tone presentation in awake animals	t-test	Pre = 0.47 ± 0.10 vs Post= 0.26 ± 0.08 (Average calcium activity $\Delta F_1/F_0$/s)	p = 0.049 (one tailed)	N = 131 neurons
S1l	Average activity of multimodal neurons in response to paired tone repeatedly presented after visual stimulus in awake animals	t-test	Pre = 0.25 ± 0.06 vs Post = 0.09 ± 0.03 (Average calcium activity $\Delta F_1/F_0$/s)	p = 0.021	N = 130 neurons
The data for Supplementary Figure 1 uses 332 recorded neurons over 12 experiments taken from six cortical regions across six animals in light anaesthetic conditions and 408 recorded neurons from four cortical regions across four animals in awake conditions.					

Supplementary Table 1. Statistical comparisons for Supplementary Figure 1, Related to Supplementary Figure 1.

<table>
<tr><th colspan="3">Statistical Comparisons
for Supplementary Figure 2c</th><th rowspan="2">Centre and dispersion or fit</th><th colspan="2">Result</th></tr>
<tr><th>Panel</th><th>Comparison</th><th>Test</th><th>p value</th><th>N value</th></tr>
<tr><td>S2c</td><td>Net GEVI response of cortical regions to tone vs visual stimulus</td><td>t-test</td><td>Tone = -0.05 ± 0.01
Grating = 0.03 ± 0.01</td><td>p < 0.001</td><td>Tone
n = 176 regions

Grating
n = 182 regions

Average of 5 trials</td></tr>
<tr><td colspan="6">The data for Supplementary Figure 2 uses 358 regions taken from five animals in light anaesthetic conditions.</td></tr>
</table>

Supplementary Table 2. Statistical comparisons for Supplementary Figure 2, Related to Supplementary Figure 2.

Statistical Comparisons for Supplementary Figure 3 a –d			Centre and dispersion or fit	Result	
Panel	Comparison	Test		p value	N value
S3a	Average correlation coefficient between multimodal neurons with different orientation preferences	MWRST	Difference in orientation preference:<br>0° = 0.178 ± 0.02<br>vs<br>90° = 0.098 ± 0.01<br>(Average correlation coefficient (r))	$p < 0.001$	93 Neurons
S3b	For increasing neurons Spontaneous activity before vs after pairing	Repeated measures ANOVA	Before = 0.54 ± 0.08<br>vs<br>After = 0.58 ± 0.08<br>(Average calcium activity $\Delta F_1/F_0$/s)	$p = 0.720$	55 Neurons
S3b	For non-increasing neurons Spontaneous activity before vs after pairing	Repeated measures ANOVA	Before = 0.49 ± 0.07<br>vs<br>After = 0.50 ± 0.09<br>(Average calcium activity $\Delta F_1/F_0$/s)	$p = 0.887$	38 Neurons
S3c	For increasing neurons with increasing network<br>vs<br>with non-increasing network During baseline in awake conditions	Repeated measures ANOVA	For increasing neurons with increasing network = 0.41 ± 0.05<br>vs<br>For increasing neurons with non-increasing network = 0.25 ± 0.02<br>(Average correlation coefficient (r))	$p < 0.001$	114 Increasing Neurons<br><br>46 Non-increasing Neurons
S3c	For non-increasing neurons with increasing network<br>vs<br>with non-increasing network During baseline in awake conditions	Repeated measures ANOVA	For non-increasing neurons with increasing network = 0.16 ± 0.02<br>vs<br>For non-increasing neurons with non-increasing network = 0.26 ± 0.04<br>(Average correlation coefficient (r))	$p = 0.036$	
S3d	Δ in network associations	t-test	Δ in association between increasing neurons with increasing network<br>5.1 ± 1.9 %<br><br>Δ in association between non-increasing neurons with increasing network<br>-2.6 ± 3.2 %<br><br>(Δ in network associations % of total correlation)	$p = 0.033$	114 Increasing Neurons<br><br>46 Non-increasing Neurons
The data for Supplementary Figure 3 uses 93 multimodal neurons taken from five cortical regions across five animals in light anaesthetic conditions and 160 neurons from four cortical regions across four animals in awake conditions.					

Supplementary Table 3. Statistical comparisons for Supplementary Figure 3, Related to Supplementary Figure 3.

Network simulation parameters	Value
r_0	1
$r_{\max}$	20
y_0	5
H_0	3
τ_θ	$1x10^{-5}$Hz
η	$1x10^{-8}$Hz
α	$2.5x10^{-9}$Hz
$w_{\max}$	0.08
$-w_{\max-\mathrm{inh}}$	1.5

Supplementary Table 4. Parameters used in the network simulation described in Figure 4, and Supplementary Figure 4.
